# Remarks on Tetanus at International Medical Congress

**Published:** 1887-10

**Authors:** J. McF. Gaston

**Affiliations:** Professor of Surgery in Southern Medical College, Atlanta, Georgia


### Southern Medical Record.

A MONTHLY JOURNAL OF PRACTICAL MEDICINE.

☞All Communications must be addressed to R. C. WORD, M. D., Managing Editor.

"*Quicquid Præcipies Esto Brevis.*"

VOL. XVII. ATLANTA, GA., OCTOBER, 1887. No. 10.

### ORIGINAL AND SELECTED ARTICLES.

### REMARKS ON TETANUS AT INTERNATIONAL MEDICAL CONGRESS.

BY J. McF. GASTON, M. D.,
Professor of Surgery in Southern Medical College, Atlanta, Georgia.

After the reading of Dr. Brown's paper on the "Etiology and Treatment of Tetanus," the President called upon Dr. J. McF. Gaston, of Atlanta, Georgia, to lead in the discussion. He regretted that the paper upon the "Microbic Oirgin of Tetanus," by Dr. Browning, had not been presented, as it doubtless would have afforded the results of his experimentation and thus given data of a definite nature upon this subject.

As the paper read before the section claimed not only the bacterial origin of the disease, but recognized it as being contagious or infectious, it behooves us to consider how far the positions taken are tenable. It has been taken for granted, in a lecture by Dr. N. Senn, that germs are the cause of tetanus, but no proof is adduced which is calculated to satisfy the investigator, and it appears in the paper presented by Dr. Brown that he had not eliminated the doubts which may exist in regard to this etiological factor.

Cases of tetanus occur within a few hours after an injury, as reported in the work of H. C. Wood, and it is not presumable that germs could enter and do their work in so brief a period. Cases result from the cicatrization of wounds in which there is no longer any abrasion or lesion to admit of their penetrating the tissues. Cases ensue from certain agents introduced into the stomach, as strychnine, so that this pathological condition of the nervous system may be induced independent of any suspicion of bacterial origin. It is begging the question to claim that tetanus is due to bacilli, because a bacillus of a distinctive character has been found in the course of the disease, since it may be an accompaniment without having any connective influence.

In regard to the claim of contagiousness based chiefly upon the observation that horses in the same locality have developed tetanus and that it has appeared synchronously or subsequently in man, it should be considered that there are predisposing atmospheric conditions in some localities, and under modifications of humidity and cold, which explain the occurrence of the disease without implying its infectiousness or its contagiousness in the ordinary acceptation of these terms.

The presumption raised in favor of its extension from animals to man under such circumstances is not tenable and more direct evidence of the communibility of this disease is requisite to secure conviction on the part of those who require facts upon which to come to a correct conclusion, and the speaker held that he could not accept such data as final.

For the proper understanding of the etiology of tetanus it is requisite to define the nature of this affection, and the pathology of the disease being involved in much doubt, the data for ascertaining the true character of tetanus are not by any means satisfactory.

All concur in pronouncing it a spasmodic disorder of the nervous system with tonic contractions of various muscles. In most cases there is not at the outset any indications of a febrile development, yet in its progress the temperature frequently rises very high and increases even after death.

The origin of the disorder is for the most part in a lesion of the remote branches of the nerves of the extremities; the hands and feet being often the seat of slight injuries which fester without forming pus, and lead to this disturbance. It is a matter of moment to determine the exact nature of the change which takes place in the injured part, but there is thus far no definite criterion for deciding the pathological status of the local trouble, and the recent researches as to ptomaines induces me to venture a solution of the difficulty upon the hypothesis of septic infection from a degeneration of the cellular elements by absorption of the decomposed and disintegrated structure of a circumscribed area, which has been injured.

Incised wounds with clean cut edges are not found to produce tetanus, but punctured or lacerated wounds are frequently the source of it, and comparatively slight abrasions of the superficial tissues may cause it.

A local necrosis of the constituents of the organization most likely results from the peculiar lesion, and there are none of the ordinary phenomena of inflammation such as heat, redness or swelling in the immediate locality, nor is the irritation of the tissues accompanied with suppuration. The local injury may or may not cause pain in the part, and, indeed, instances have occurred in which the individual is not conscious of having received a wound, so that it becomes necessary to search for the peripheral lesions after the spasm of the muscles is developed.

It is a mooted point, whether tetanus ever occurs independent of a local lesion of the peripheral fibrillæ of the nerves in some portion of the body. Most of the so-called idiopathic cases are referable upon close investigation to a wound not previously recognized by the patient and doubtless there may in some instances exist internal lesions which are not capable of being discerned by any mode of examination externally.

What is known of the etiology of tetanus? Will any one claim that the proximate cause of the disease is understood or that its pathology has been accurately defined? Certainly no one who is acquainted with the observations of those most competent to interpret what is found after death from tetanus, either in cases which run their course to a fatal result in a few hours, or those which have terminated in death after days and weeks of suffering, can feel assured of the exact process which had brought about the result.

We have instances recorded in which death has ensued from tetanus in a very brief space of time after the infliction of a comparatively slight injury superficialy, when it was simply impossible that any inflammatory process could have extended from the peripheral to the central nervous organization.

It would seem impossible under such conditions that germs could have any agency in developing the disease, and though a bacillus is found in the progress of some protracted cases, it is not likely that it enters as an etiological factor. The recourse to germs in lieu of other causes is like the case of a man who recommended his dog for coons, because he had never proved useful for anything else. If we proceed by exclusion to settle down upon the bacterial origin of tetanus, it has no real foundation.

As to the contagiousness of the disease all may be explained upon the existence of a common origin.

The theory of neuritis being extended to the spinal column and producing a modified myelitis does not seem a satisfactory solution of the perturbation of the nervous system and the muscular organization which characterize this disease.

The view of reflex action through the spinal column does not meet the developments presenting tonic spasm of the muscles. There is a relation between the minute nerves and capillaries, which is transmitted to the nerve centres of the ganglionic and spinal systems, that may give a clue to the phenomena of tetanus, and this correlation has been defined in a paper on the pathology and therapeutics of the diseases of the nerve centres, etc., presented by me to tne Medical Association of Georgia and published with the transactions of 1884.

If we can appreciate properly the changes which ensue in the part injured and comprehend the propagation of this septic contamination from the peripheral to the central portion of the nervous system, we may have an interpretation of the grave results ensuing from an apparently slight cause.

That it does not partake of the nature of lymphangitis, nor present any of the characteristics of ordinary inflammatory action in the local lesion, seems to exclude all the agencies which operate in producing such changes and leaves us to study the element of nervous irritation separately.

In reference to the treatment of tetanus, all concurred, perhaps, in the general management of these cases adverted to in the paper, upon a recognition of those agencies which operated through the nervous system.

The attention of the section was asked to the results obtained by the speaker in securing favorable results in ten out of eighteen cases which had come under his observation in this country and in South America. It had been his practice to obtain the controlling effects of chloroform at the outset. If there appeared any marked derangement of secretions remedies were then applied for their correction, and afterwards a recourse to bromide of potash and chloral hydrate until their effects were manifested profoundly had been attended with salutary influence upon the tetanic spasms. Morphine with quinine had been resorted to in the intervals with good results. It was found in one case that the lobelia inflata given by the mouth and by enemata, when the stomach would not retain the remedy, had secured a cure in a well defined case of traumatic tetanus. This case was referred to in *Wood & Beaches'*

*Dispensatory* many years ago under the head of lobelia. Tobacco injections had also been efficacious in a case, but attended with such depression of the vital powers as to cause serious apprehension of a fatal prostration, and to deter him from its repetition in other cases.

The local application of chloroform to the spinal column had been resorted to in accordance with the inhibitory action of such an application noted by Brown-Sequad in the inferior animals, but no ultimate advantage had been gained by this local measure.

A report of the treatment of tetanus by the hypodermic injection of chloral into the point of the lesion had been made by some author, whose name could not be recalled, with complete relief in a case of tetanus. This seemed a rational mode of proceeding as the remedial agent went out into the system through the same channel that the disease entered, and was certainly entitled to the consideration of those who would proceed upon phylosophical principles in treating this affection.

The general good results obtained from saturating the subject with a combination of bromide of potash and the chloral hydrate, so as to produce a decided impression, affords encouragement for the most satisfactory result and is confidently commended.

